# A CT-based radiomics nomogram for predicting prognosis of coronavirus disease 2019 (COVID-19) radiomics nomogram predicting COVID-19

**DOI:** 10.1259/bjr.20200634

**Published:** 2020-12-09

**Authors:** Hang Chen, Ming Zeng, Xinglan Wang, Liping Su, Yuwei Xia, Quan Yang, Dan Liu

**Affiliations:** 1 Department of Radiology, The Yongchuan Hospital of Chongqing Medical University, Chongqing, China; 2 Department of Respiratory and Critical Care Medicine, The Yongchuan Hospital of Chongqing Medical University, Chongqing, China; 3 Huiying Medical Technology Co., Ltd, Dongsheng Science and Technology Park, Beijing, China

## Abstract

**Objectives:**

To identify the value of radiomics method derived from CT images to predict prognosis in patients with COVID-19.

**Methods:**

A total of 40 patients with COVID-19 were enrolled in the study. Baseline clinical data, CT images, and laboratory testing results were collected from all patients. We defined that ROIs in the absorption group decreased in the density and scope in GGO, and ROIs in the progress group progressed to consolidation. A total of 180 ROIs from absorption group (*n* = 118) and consolidation group (*n* = 62) were randomly divided into a training set (*n* = 145) and a validation set (*n* = 35) (8:2). Radiomics features were extracted from CT images, and the radiomics-based models were built with three classifiers. A radiomics score (Rad-score) was calculated by a linear combination of selected features. The Rad-score and clinical factors were incorporated into the radiomics nomogram construction. The prediction performance of the clinical factors model and the radiomics nomogram for prognosis was estimated.

**Results:**

A total of 15 radiomics features with respective coefficients were calculated. The AUC values of radiomics models (kNN, SVM, and LR) were 0.88, 0.88, and 0.84, respectively, showing a good performance. The C-index of the clinical factors model was 0.82 [95% CI (0.75–0.88)] in the training set and 0.77 [95% CI (0.59–0.90)] in the validation set. The radiomics nomogram showed optimal prediction performance. In the training set, the C-index was 0.91 [95% CI (0.85–0.95)], and in the validation set, the C-index was 0.85 [95% CI (0.69–0.95)]. For the training set, the C-index of the radiomics nomogram was significantly higher than the clinical factors model (*p* = 0.0021). Decision curve analysis showed that radiomics nomogram outperformed the clinical model in terms of clinical usefulness.

**Conclusions:**

The radiomics nomogram based on CT images showed favorable prediction performance in the prognosis of COVID-19. The radiomics nomogram could be used as a potential biomarker for more accurate categorization of patients into different stages for clinical decision-making process.

**Advances in knowledge:**

Radiomics features based on chest CT images help clinicians to categorize the patients of COVID-19 into different stages. Radiomics nomogram based on CT images has favorable predictive performance in the prognosis of COVID-19. Radiomics act as a potential modality to supplement conventional medical examinations.

## Introduction

A group of patients with pneumonia of unknown aetiology were first reported in Wuhan, Hubei, China, on December 8, 2019. On February 11, 2020, the disease was named coronavirus disease 2019 (COVID-19) by the World Health Organization (WHO). With the gradual recognition of COVID-19 pneumonia, especially in severely ill patients, professional consensus and guidelines were developed to prevent transmission and promote diagnosis and therapy.^1–3^ Chest imaging has shown to be very useful in diagnosing severe COVID-19 cases with respiratory symptoms.^4^

Recent literature summarized the CT manifestations and dynamic changes of COVID-19, which reported that the typical CT findings of COVID-19 showed a bilateral distribution of ground-glass opacities (GGO) with or without consolidation in posterior and peripheral lungs. These features are similar to the ones observed in other coronavirus infections.^4–6^ However, some CT imaging findings were not so typical.^7,8^ Chung et al^6^ reported that GGO might appear in most patients, and thus, is considered as the earliest radiographically visible CT manifestation in some patients. A previous study showed that lung involvement gradually increased to consolidation up to 2 weeks after the onset of initial symptoms.^9^ The preliminary study by Fang et al^5^ showed that GGO could be observed to demonstrate the consolidation absorption. Consolidation has been considered as an indication of disease progression that serves as an alert in the management of patients.^10^ Early prediction based on CT imaging of the whole disease process of COVID-19 could prompt early clinical diagnosis, speed-up treatment and early isolation, providing evidence for evaluating the effect of a comprehensive therapy.

The purpose of this study was to predict the prognosis of COVID-19 and help the physicians to more accurately categorize patients into different stages so as to provide better decision-making for patients, such as the need for ICU, length of hospital stay, and the requirement for oxygen.

## Methods and materials

### Patients and clinical factors

The institutional review board approved the study, and patient informed consent was waived for this retrospective analysis.

A total of 40 patients who were positive for COVID-19 between 21 January and 26 March 2020 were enrolled in the study. COVID.19 was confirmed by laboratory testing of respiratory secretions obtained by bronchoalveolar lavage, endotracheal aspirate, nasopharyngeal swab, or oropharyngeal swab. Specific inclusion criteria were: (1) patients with a positive new coronavirus nucleic acid antibody admitted to our hospital; (2) patients who underwent CT after admission; and (3) CT images confirmed the presence of pneumonia. Exclusion criteria were: (1) patients who did not have GGO in the first CT examination; (2) patients without significant changes of lesions in the repeat CT after 5–7 days; and (3) lesions with consolidation on the initial CT. Baseline clinical data were collected and included age, sex, severity, symptoms, travel and exposure history, as well as laboratory testing results. Univariate analysis was used to identify the correlation between clinical factors, radiomics features, and radiological progression. A multiple logistic regression analysis was applied to develop the clinical factors model by using the significant variables from the univariate analysis as inputs. Correlation coefficients (r) with its 95% confidence interval (CI) were calculated for each independent factor.

### CT protocol

Chest CT scans were performed using a single inspiratory phase and non-enhanced scanning in the commercial multidetector CT scanner (256-section Philips Brilliance iCT). CT images were acquired during a single breath-hold. The CT protocol was as follows: volume scan, tube voltage of 120 kVp with automatic tube current modulation, slice thickness, and interval of 5 mm. The same CT protocol and scanner were applied to the two CT scans. The fourth-generation iterative reconstruction (IR) algorithm (iDose4) and sharp kernel were applied to proceed the thin-slice (1 mm) reconstruction.

### Radiomics workflow

**Figure 1.**
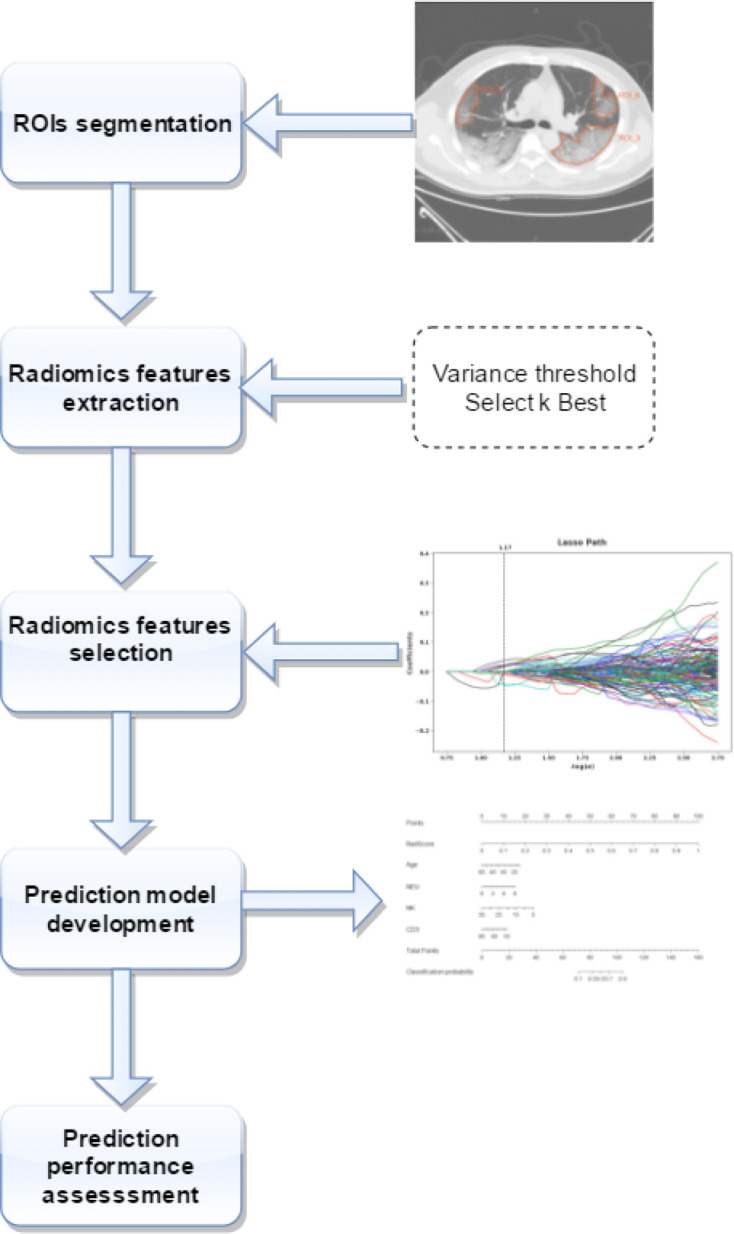
Radiomics workflow.

Figure 1 presents the radiomics workflow, including (1) ROIs segmentation, (2) radiomics features extraction, (3) radiomics features selection, (4) prediction model development (in training set), and (5) prediction performance assessment.

### ROIs segmentation

All the images were exported from the PACS system and imported into a radiomics cloud platform V.3.1.0 (http://radcloud.cn/, Huiying Medical Technology Co., Ltd, Beijing, China). The lesions were manually delineated on the reconstructed images with a slice of 1 mm by two independent radiologists (XLW and DL with approximately 18 and 15 years of experience in thoracic radiology, respectively). CT manifestations of COVID-19 may vary among different patients and stages; thus, we took each lesion as a unit to perform radiomics analysis and segmented the regions of interest (ROIs). We set up the prerequisites that all ROIs were GGO; after which patients underwent a second CT examination (the time interval between the two CT scans was 5–7 days). Three experienced radiologists (XLW, LPS, and QY) evaluated the progression conditions of COVID-19 comparing the two CT images dependently and were blinded to the clinical information.

**Figure 2.**
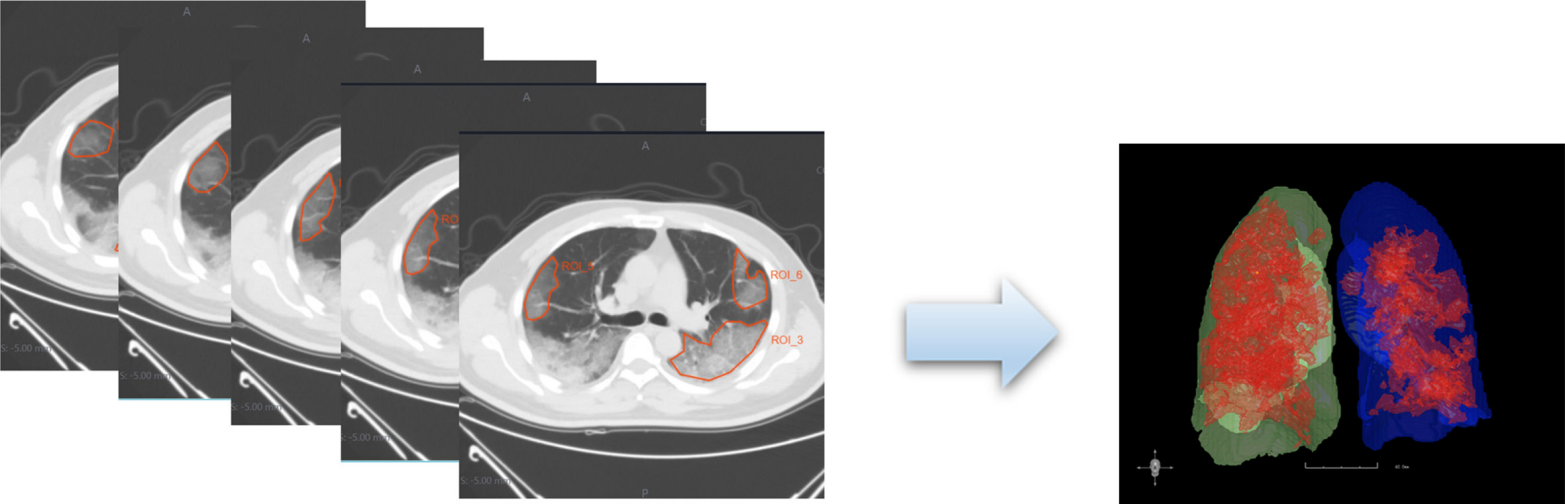
An example of the manual segmentation in lesions of COVID-19. Manual segmentation on the same axial slice.

An example of the manual segmentation is shown in Figure 2. GGO was defined as a hazy increase in lung attenuation with no obscuration of the underlying vessels, which was also manifestation in the absorption period of COVID-19.^8^ Consolidation was considered as an indication of disease progression. After CT re-examination, ROIs were finally divided into two groups based on the condition of radiological progression: absorption group and consolidation (progression) group. ROIs in the absorption group decreased in the density and scope in GGO, and ROIs in the progress group progressed to consolidation. Inter- and intra-class correlation coefficients (ICCs) were used to assess the intraobserver reproducibility and inter observer reliability of feature extraction. There was a good agreement of the feature extraction if the ICC value <0.75.

### Radiomics feature extraction and selection

The radiomics features were divided into four groups: (a) a first-order statistics, including 126 descriptors that quantitatively delineated the distribution of voxel intensities within ROIs through commonly used and basic metrics; (b) shape features, composed of three-dimensional (3-D) features that reflected the shape and size of the region; (c) textural features, which were calculated from Grey Level Run-Length Matrix (GLRLM), Grey Level Co-occurrence Matrix (GLCM),Gray Level Size Zone Matrix (GLSZM) and Gray Level Dependence Matrix(GLDM); (d) filter and wavelet features, which included the intensity and texture features derived from filter transformation and wavelet transformation of the original images, processed using filters, such as wavelet-LLL, wavelet-LHL, wavelet-HLL, wavelet-LLH, logarithm, square, square root, original, and exponential.^11,12^

In the present study, three methods were used to progressively reduce the redundant features. Firstly, the variance threshold method was applied to remove the eigenvalues of the variance smaller than 0.8. Secondly, the Select K Best method by using *p* value to analyze the correlation between the features and the classification results; the selected features were used (*p* < 0.05). Finally, the least absolute shrinkage and selection operator (LASSO) model were used to reduce the dimensions of features and effectively identify the most significant features.^13,14^ For the LASSO model, the error value of cross-validation was 10, and the maximum number of iterations was 2000. The corresponding parameter settings followed the previous studies.^15–17^

### Classification analysis and radiomics signature

Classification analysis was performed to identify absorption and consolidation based on texture features based on CT images. The classifiers, which were constructed by supervised learning, involved learning from a cluster of given samples possessing the selected features so as to create a classifier that can correctly classify new objects and predict the data with respect to prognosis.^18^ In this study, the radiomics-based models were built with three classifiers, k-Nearest Neighbor (kNN), Support Vector Machine (SVM), and Logistic Regression (LR). The decision tree algorithm first creates readable rules and decisions by using an inductive algorithm; then, this decision is used to analyze new data.^19^ The prediction performance of the radiomics-based classifiers for predicting the progress of COVID-19 was assessed concerning the area under the curve (AUC), the receiver operating characteristic (ROC) curve, sensitivity, specificity, and accuracy in the training and validation sets. The selected features were operated to build radiomics signature, and a radiomics signature (Rad-score) was calculated by a linear combination of selected features weighted by corresponding LASSO coefficients.^20^

### Development of a radiomics nomogram and performance assessment

The significant variables of both the clinical factors and the Rad-score were employed to develop a radiomics nomogram. A calibration plot was performed to assess the calibration and goodness-of-fit of the nomogram. The prediction performance of the clinical factors model and the radiomics nomogram for prognosis was estimated based on C-index in both the training and validation sets. The decision curve analysis (DCA) was conducted to assess the net benefits for a range of threshold probabilities in the training set.

### Statistical analysis

Statistical analyses were performed using SPSS v.24.0 (SPSS Inc., Chicago, IL, USA) and R statistical software v.3.3.4 (https://www.r-project.org). Between-group comparisons of the clinical factors were conducted with the chi-squared test or Fisher exact test for categorical variables, the continuous variables were conducted with the Mann-Whitney U-test. The ROCs of the two models were compared using the DeLong test. The prediction performance of models was assessed in the validation set by the same thresholds determined in the training set. The ROC curves were plotted using the “pROC” package. Nomogram development was conducted by using the “rms” package. The DCA was performed using the “dca.R.” package.

## Results

### Clinical information and clinical factors model

Forty patients were included in our study (age: 47.6 ± 14); 25 (62.5%) were males, and 18 (45%) were previously exposed to COVID-19. The detailed clinical data of patients are summarized in Table 1. Table 2 shows the relationship between the clinical factors, including T lymphocyte subgroups, and the radiological progression of patients with COVID-19. There was significant difference in age, neutrophil count, NK cells %, NK cells count, CD3^+^T%, CD19^+^%, and CD4^+^T% between the absorption group and consolidation group (*p* < 0.05); but CD19^+^ count, CD3^+^CD8^+^T%, CD3^+^T count, C-reactive protein (CRP), sex, the ratio of CD4^+^/CD8^+^, CD4^+^ T count, CD8^+^ T count were not significantly different (*p* > 0.05). Table 3 shows *p* values of clinical information corresponding to ROIs in the training and validation sets.

**Table 1.** Demographic and clinical characteristics of 40 patients with COVID-19

Characteristic		Number (%)
Sex	Male	25 (62.5%)
	Female	15 (37.5%)
Age		47.625 ± 14
COVID-19 exposure history		18 (45%)
Signs and symptoms		
Fever	Higher (＞37.3°C) temperature	23 (57.5%)
	Normal(36.3℃−37.3℃)	17 (42.5%)
Cough		16 (40%)
Little phlegm		12 (30%)
Myalgia or fatigue		5 (12.5%)
Mild dyspnea or chest pain		3 (7.5%)
Mild dizziness		1（2.5%）
Loss of appetite		1（2.5%）
Diarrhea		1（2.5%）
Stuffy and runny nose		1（2.5%）
Sore throat		1（2.5%）
Nausea and vomit		1（2.5%）

**Table 2.** The correlation between clinical factors and radiological progression of COVID-19 patients

	Radiological progression		*p*
	r	95% CI	
Age	−0.3858	−0.5039–0.2534	<0.0001
Sex	0.0940	−0.0535–0.2374	0.2109
Neutrophil count	0.3056	0.1663–0.4328	<0.0001
CD19^+^%	0.2017	0.0567–0.3383	0.0068
CD19^+^ count	0.0619	−0.0856–0.2067	0.4106
CD3^+^T%	0.1770	0.0312–0.3155	0.0178
CD3^+^CD8^+^T%	0.0780	−0.0694–0.2222	0.2992
CD3^+^T count	0.0813	−0.0662–0.2253	0.2793
CRP	−0.0938	−0.2372–0.0537	0.2119
NK cells count	−0.2086	−0.4104–0.1396	0.0001
NK cells%	−0.3718	−0.4916–0.2318	<0.0001
CD4^+^/CD8^+^	0.0746	−0.0728–0.2189	0.3207
CD4^+^T%	0.1821	0.0364–0.3202	0.0147
CD4^+^ T count	0.1466	−0.0001–0.2871	0.0503
CD8^+^ T count	0.0470	−0.1004–0.1923	0.5324

**Table 3.** Clinical information corresponding to lesions in the training and validation cohorts

Clinical information	Training cohort （*n* = 145）	Validation cohort (*n* = 35)	*p*
Age (yrs, mean ± SD)	50.2 ± 12.5	50.5 ± 14.8	0.900
Gender			
Male	111	30	0.240
Female	34	5	
Clinical factors			
NEU count (mean ± SD)	3.7 ± 1.6	4.3 ± 2.0	0.042
NK (%)	22.2 ± 8.5	24.1 ± 9.6	0.267
CD3 (%)	60.2 ± 12.6	56.5 ± 14.6	0.125

### Radiomics feature and prediction performance of radiomics-based models

**Figure 3.**
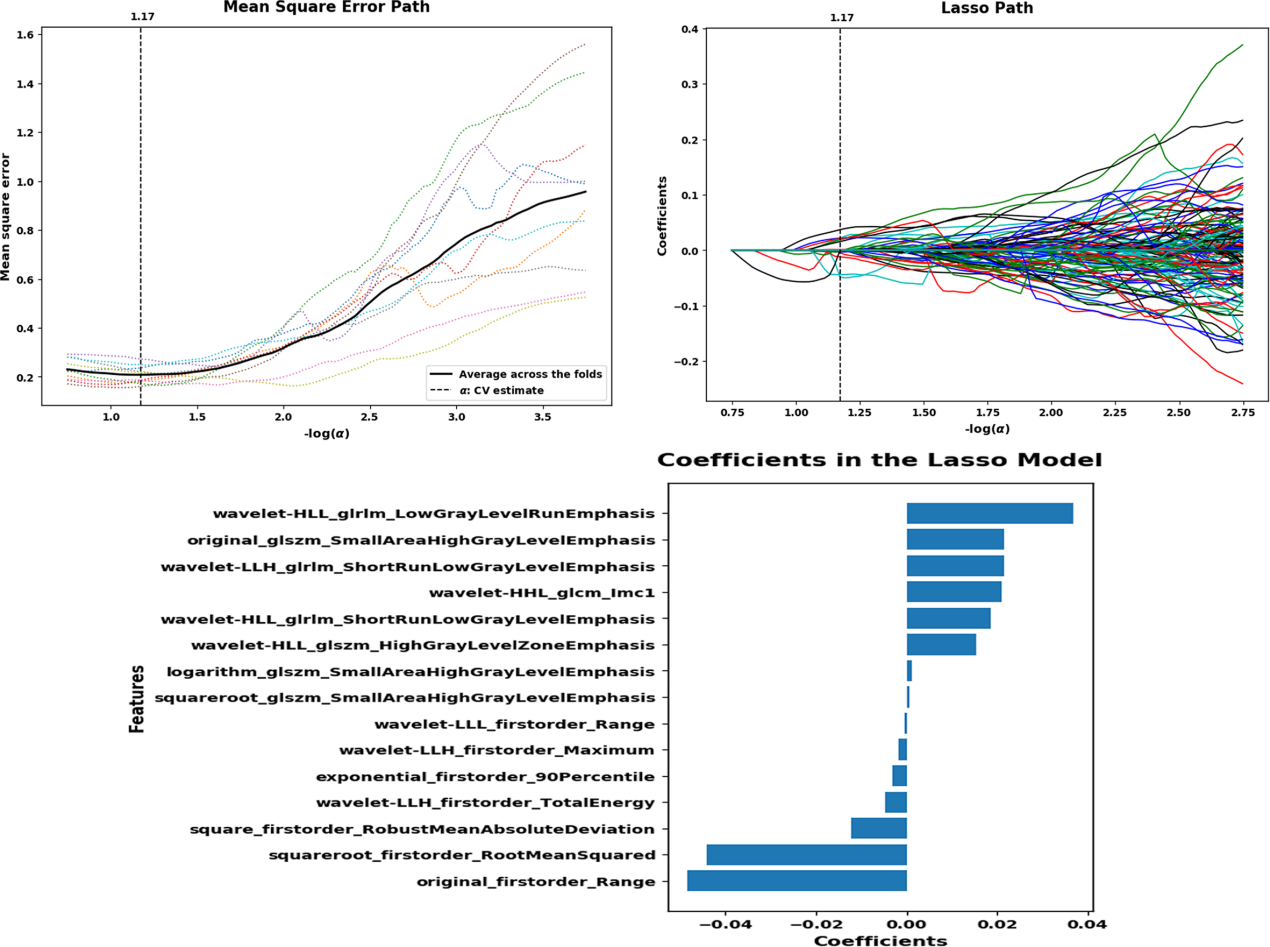
LASSO regression model on CT images. Mean square error on each fold in tenfold cross-validation method and the optimal value of the lasso tuning parameter (α = 1.17) is found. And 15 features which are correspond to the optimal α value are extracted following coefficients.

A total of 180 ROIs were analyzed; 1409 image features were extracted from the CT images. ROIs from the absorption group (*n* = 118) and consolidation group (*n* = 62) were randomly divided into a training set (*n* = 145) and a validation set (*n* = 35). For the selection of radiomics features, significant features were selected by the LASSO regression model and forward selection approach (Figure 3). The best performance of LASSO regression was built using a penalty parameter α = 1.17, as the mean square error was minimized. Finally, 15 radiomics features with respective coefficients were calculated (Table 4).

**Table 4.** Lasso coefficients of 15 radiomics features

Features	Coefficients
original_firstorder_Range	−0.04843
squareroot_firstorder_RootMeanSquared	−0.04421
square_firstorder_RobustMeanAbsoluteDeviation	−0.01221
wavelet-LLH_firstorder_TotalEnergy	−0.00483
exponential_firstorder_90Percentile	−0.00308
wavelet-LLH_firstorder_Maximum	−0.00179
wavelet-LLL_firstorder_Range	−0.00047
squareroot_glszm_SmallAreaHighGrayLevelEmphasis	0.000629
logarithm_glszm_SmallAreaHighGrayLevelEmphasis	0.001141
wavelet-HLL_glszm_HighGrayLevelZoneEmphasis	0.015355
wavelet-HLL_glrlm_ShortRunLowGrayLevelEmphasis	0.018665
wavelet-HHL_glcm_Imc1	0.020934
wavelet-LLH_glrlm_ShortRunLowGrayLevelEmphasis	0.021557
original_glszm_SmallAreaHighGrayLevelEmphasis	0.021587
wavelet-HLL_glrlm_LowGrayLevelRunEmphasis	0.036865

**Figure 4.**
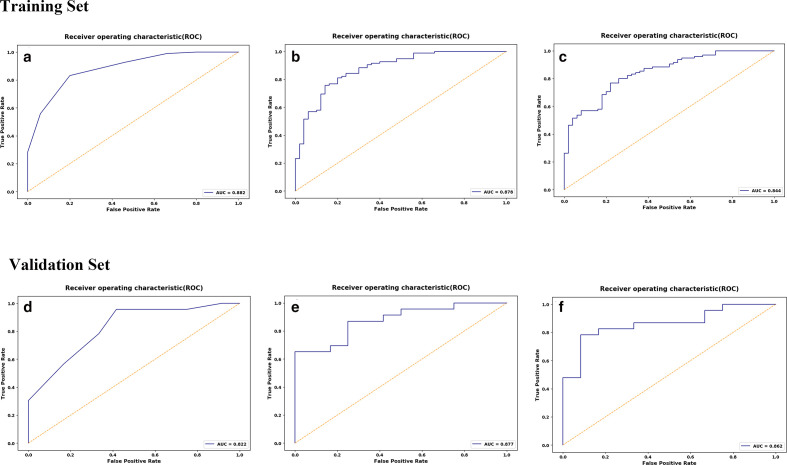
ROC curves of the kNN(A), SVM(B), LR(C) classifiers in the training set. ROC curves of the kNN(D), SVM(E), LR(F) classifiers in the validation set.

ROC analysis was used to evaluate the prediction performance of radiomic-based models (Table 5). In the training set, the accuracy, sensitivity, specificity, and AUC of kNN, SVM and LR were 0.82, 0.83, 0.80, 0.88; 0.79, 0.76, 0.86, 0.88; 0.77, 0.77, 0.78, 0.84, respectively. In the validation set, the overall accuracy, sensitivity, specificity, and AUC of kNN, SVM and LR were 0.83, 0.96, 0.58, 0.82; 0.77, 0.65, 1.0, 0.88; 0.83, 0.78, 0.91, 0.86, respectively (Figure 4). With regard to accuracy, the kNN demonstrated the best performance among the three models. AUC values under ROCs of multiple radiomics models obtained by a tenfold cross-validation method showed better performance.

**Table 5.** The results of radiomics analysis for classifications

Classifiers	Radiological progression	Training set					Validation set				
		ACC	SEN	SPE	AUC	Cut-off	ACC	SEN	SPE	AUC	Cut-off
kNN	Absorption	0.82	0.83	0.80	0.88	0.71	0.83	0.96	0.58	0.82	0.57
	Consolidation										
SVM	Absorption	0.79	0.75	0.86	0.88	0.72	0.77	0.65	1.0	0.88	0.73
	Consolidation										
LR	Absorption	0.77	0.76	0.78	0.84	0.50	0.83	0.78	0.92	0.86	0.51
	Consolidation										

ACC, Accuracy; AUC, Area under the curve; LR, Logistic regression; SEN, Sensitivity; SPE, Specificity; SVM, Support vector machine;kNN, k-Nearest neighbor.

### Radiomics signature construction

The radiomics signature was developed by 15 features. The Rad-score was calculated using the following formula:

Rad-score = 0.6552 – 0.0484 × original_firstorder_Range – 0.044

- × squareroot_firstorder_RootMeanSquared – 0.0122
- × square_firstorder_RobustMeanAbsoluteDeviation – 0.0048
- × wavelet-LLH_firstorder_TotalEnergy – 0.0031
- × exponential_firstorder_90Percentile – 0.0018
- × wavelet-LLH_firstorder_Maximum – 0.0011
- × logarithm_glszm_SmallAreaHighGrayLevelEmphasis + 0.0006
- × squareroot_glszm_SmallAreaHighGrayLevelEmphasis + 0.0011
- × logarithm_glszm_SmallAreaHighGrayLevelEmphasis + 0.0153
- × wavelet-HLL_glszm_HighGrayLevelZoneEmphasis + 0.0187
- × wavelet-HLL_glrlm_ShortRunLowGrayLevelEmphasis + 0.0209
- × wavelet-HHL_glcm_Imc1 + 0.0216
- × wavelet-LLH_glrlm_ShortRunLowGrayLevelEmphasis + 0.0216
- × original_glszm_SmallAreaHighGrayLevelEmphasis + 0.0369
- × wavelet-HLL_glrlm_LowGrayLevelRunEmphasis

The Rad-score showed significant difference between absorption and consolidation (*r* = 0.5022, 95% CI:0.3838–0.6044, *p* < 0.0001).

### Radiomics nomogram construction and prediction performance assessment

**Figure 5.**
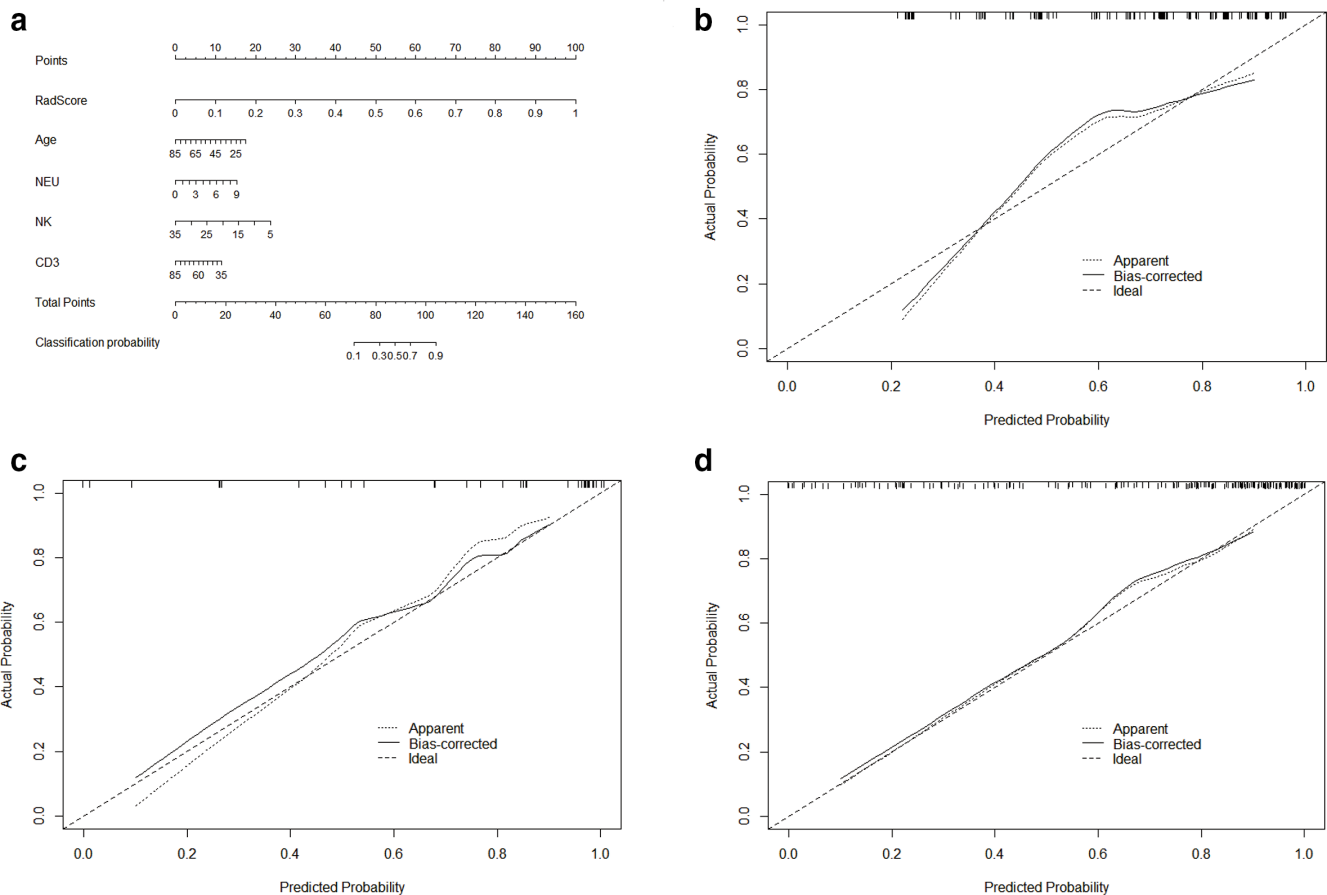
The radiomics nomogram and calibration curves for the radiomics nomogram. (a)The radiomics nomogram, combining Rad-score, age, neutrophil count, NK% and CD3^+^%, built in the training set. Calibration curves for the radiomics nomogram in the training (b), validation (c) sets and the whole cohort (d). Calibration curves indicate the goodness-of-fit of the nomogram. The 45° dotted line represents the ideal prediction, and the bias-corrected solid line represents the predictive performance. The closer the solid line approaches the ideal prediction line, the better the predictive efficacy of the nomogram is.

**Figure 6.**
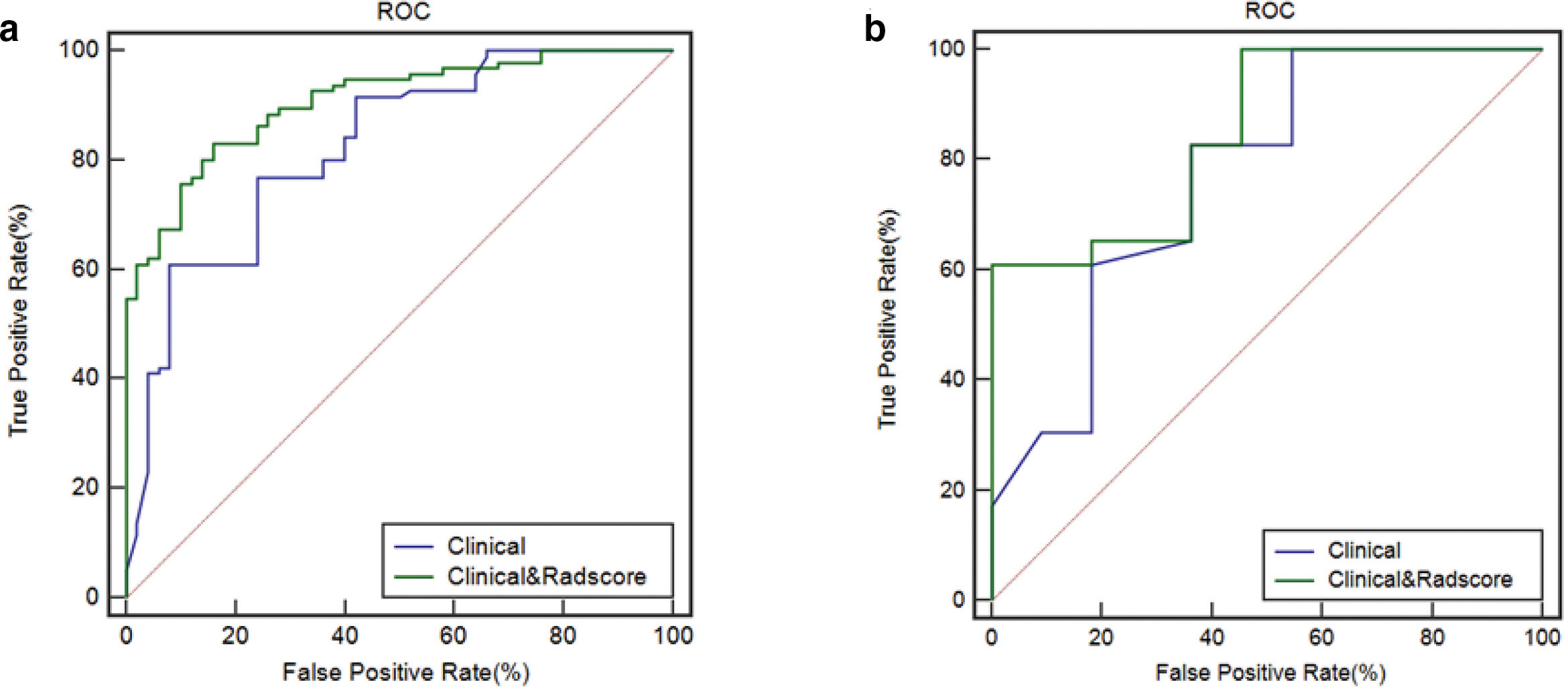
The ROC curves of the clinical factors model and the radiomics nomogram in the training (a) and validation sets (b). The C-index of the radiomics nomogram was significantly higher than clinical factors model in the training set.

**Figure 7.**
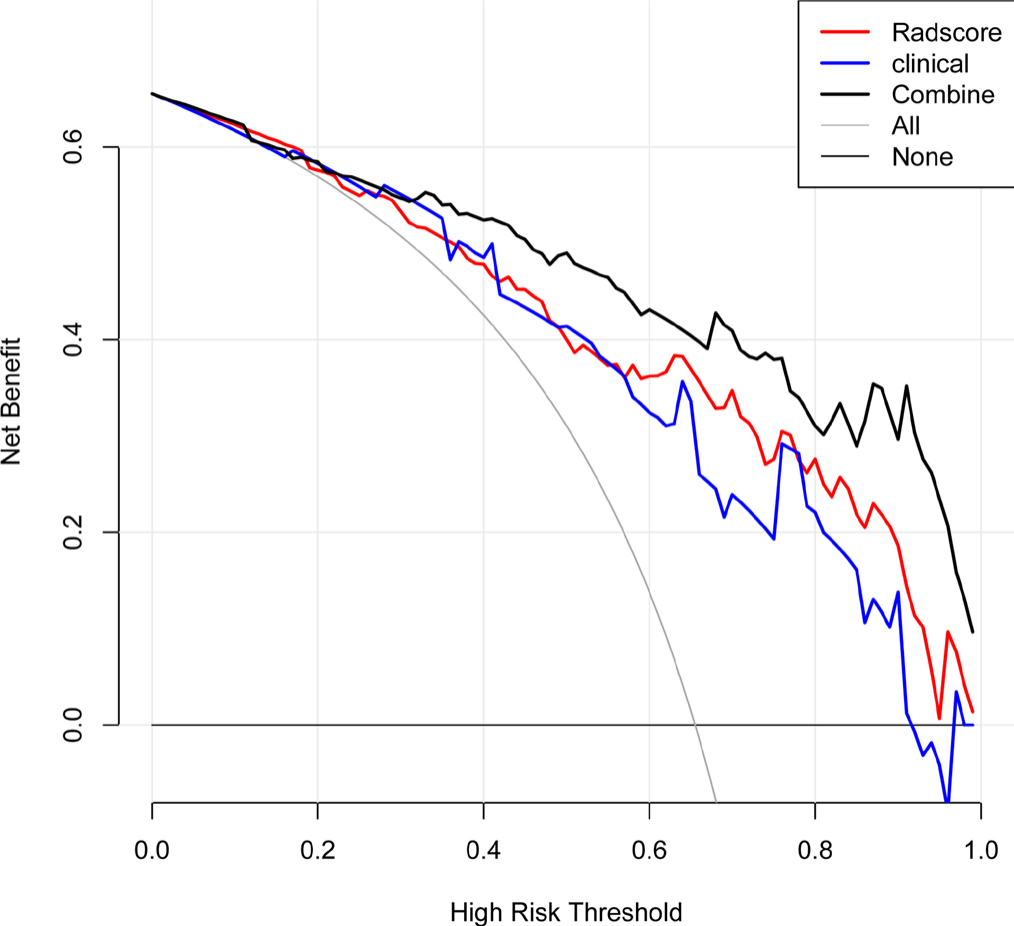
Decision curve analysis for the radiomics nomogram, the clinical factor, and the radiomics signature. The x-axis, y-axis indicate threshold probability and the net benefit, respectively. The black line, red line, and clinical line represent net benefit of the radiomics nomogram, the clinical factor, and the radiomics signature, respectively. The radiomics nomogram showed the highest net benefit in the three models.

The Rad-score, age, neutrophil count, NK%, and CD3% were incorporated into the radiomics nomogram construction (Figure 5a). Calibration curves for the radiomics nomogram in the training, validation sets, and the whole cohort are shown in Figure 5b–d. The ROC analysis was used to evaluate the prediction performance of the clinical factors model and the radiomics nomogram. We found that when the clinical factors were used for predicting prognosis, the C-index was 0.82 [95% CI (0.75–0.88); sensitivity, 0.61; and specificity, 0.92] in the training set, and 0.77 [95% CI (0.59–0.90); sensitivity: 0.83; specificity: 0.64] in the validation set. In comparison, the radiomics nomogram showed optimal prediction performance. In the training set, the C-index was 0.91 [95% CI (0.85–0.95); sensitivity: 0.83; specificity: 0.84], and the C-index was 0.85 [95% CI (0.69–0.95); sensitivity: 0.61; specificity: 1.0] in the validation set. Figure 6 showed the ROC curves of the clinical factors model and the radiomics nomogram in the training and validation sets, respectively. For the training set, the C-index of the radiomics nomogram was significantly higher than clinical factors model (*p* = 0.0021). The calibration curve showed good calibration in the training set and validation set. The results of DCA in the training set are shown in Figure 7. The radiomics nomogram showed the highest net benefit in the three models.

## Discussion

The purpose of this study was to identify the value of radiomics derived from CT images to predict prognosis in patients with COVID-19. The results showed that the radiomics nomogram integrating Rad-score and clinical factors has a good predictive value for radiological progression.

Laboratory testing has a vital role in diagnosing and managing human pathologies, including COVID-19.^21^ Reverse transcription-polymerase chain reaction (RT-PCR) on respiratory tract specimens is considered the gold standard for the etiological diagnosis of COVID-2019 infection.^22,23^ Still, according to recent reports, the diagnostic accuracy of RT-PCR testing for COVID-19 might be lower than optimal.^24^ Ai et al^25^ examined 1014 patients suspected of having COVID-19 who underwent RT-PCR testing and chest CT scan. They found that the diagnostic accuracy of the CT was higher than the RT-PCR (88% of patients had positive chest CT findings while only 59% were positive on RT-PCR). Moreover, recent study reported the combination of RT-PCR (real-time) with clinical symptoms, epidemiological evidence, and CT manifestations facilities diagnosis of COVID-19.^26^

Guan et al^27^ investigated the clinical characteristics of patients with COVID-19 in China and reported that the majority of cases on admission presented with lymphocytopenia, thrombocytopenia, and leukopenia. Most of the patients had elevated levels of CRP. Fever and cough were the dominant symptoms, while gastrointestinal symptoms were uncommon.^28,29^

A series of clinical factors were enrolled in this study. We found that the consolidation group had higher age, elevated neutrophil count, and changes of T lymphocyte subsets compared with the absorption group, which was consistent with previous studies. In general, during the early phase of the COVID-19 infection, the diagnosis and evaluation were complicated by the diversity in symptoms and imaging findings, and in the severity of disease at the time of presentation.

The chest CT has great significance in diagnosing, monitoring progression, and evaluating curative effect in clinic; yet, the role of CT for COVID-19 diagnosis and conditional evaluation is still controversial. So far, only a few studies reported in detail CT features commonly found in COVID-19.^3,6,30^ GGO and consolidation are two main manifestations of COVID-19 lesions on chest CT. Li et al^31^ reported singular or multiple irregular lesions of GGO or/and consolidation in 49 out of the 51 cases who underwent chest CT (96.1%). In this study, the CT findings were GGO in the early stage; some patients’ lesions were absorbed, and the density in lesions gradually decreased. In contrast, in some patients with disease progression, GGO turned into consolidation, and the density in the lesions increased. In this regard, CT could be able to predict what patients may progress, then taking early intervention to partly reduce the incidence of severe COVID-19 and improve the prognosis of patients (*e.g.* micronutrient, antiviral treatment and immunotherapy). However, chest CT is still limited when identifying specific viruses. The CT features of COVID-19 overlap with the features of diseases caused by viruses from a similar family, such as MERS-CoV or SARS-CoV. Moreover, these findings were qualitative, which limited their accuracy. Therefore, new ways for evaluating CT features for predicting disease progression that could guide the clinical therapies are urgently needed.

Recently, radiomics has been proven to be a potential imaging modality to identify biological characteristics of diseases beyond visual assessment on CT images. A previous study applied radiomics-based predictive models using random forest (RF) and kNN classifiers to identify glucocorticoid-sensitive connective tissue disease-related interstitial lung disease, obtaining AUC of 0.66 in RF models and 0.61 in kNN model.^32^ Zhang et al^33^ incorporated CT-radiomics and PET metabolic parameters to build a classification model (using a SVM method) for distinguishing benign and malignant lung lesions. Their model showed a substantial diagnostic capacity.

According to our knowledge, this study first reported on CT-based radiomics in prognosis prediction of COVID-19. We applied three classifiers, including kNN, SVM, and LR to develop radiomics-based models to predict absorption and consolidation of lesions. Our results revealed that all the three models had good predicting performance in feature classification methods (accuracy >0.70, AUC >0.80). Adequate analysis of clinical factors and imaging findings is helpful for accurate diagnosis and management of patients with COVID-19. Then, Rad-score, which was calculated to combine with independent clinical factors to build a radiomics nomogram, achieved favorable efficacy in predicting radiological progression in lesions of COVID-19. Additionally, the nomogram with Rad-score had a relatively higher C-index, and net benefit than the clinical factors model did, suggesting the additional value of the CT texture features in differentiating absorption group and consolidation group. Radiomics, as a computer-assisted technique, helps to identify microscopic features associated with the biology of disease progression. Assessment based on clinical factors separately cannot be used to fully evaluate the course of pneumonia; integrating the radiomics features, and clinical factors within a combined nomogram could achieve earlier detection of higher risk patients. Although chest radiographs are mainly used for COVID-19 management worldwide due to several reasons (radiation dose, patient transport, efficiencies, availability, etc),^34,35^ the prediction performance based on radiomics features can be hardly achieved with chest radiographs due to the limit on the number of images, which lead to the loss of image information.

This study has a few limitations. First, it is a retrospective cohort study; thus, potential selection bias might influence the repeatability and stability of the results. Patients who were already in the hospital and who underwent CT had either more serious clinical conditions or rather an atypical one. Second, a limited number of patients were included in the study, which may influence the generalizability of the final conclusion. Third, in a few patients, simultaneous consolidation lesions were observed, but relatively few in the early stage of the disease that had a small overall effect on results. Finally, the present study did not discuss the fibrosis condition. Still, some radiologists hinted that fibrosis might indicate a poor outcome of COVID-19, reporting that it may subsequently progress to peak stage or result in pulmonary interstitial fibrosis disease.^9,36^ The sample size should be expanded and included in the fibrosis group for prognostic evaluation.

## Conclusion

The radiomics nomogram based on CT images has favorable prediction performance in the prognosis of COVID-19. The radiomics nomogram, as a quantitative and noninvasive modality, could act as a potential biomarker to supplement conventional imaging and laboratory examinations and help physicians to more accurately categorize patients into different stages for clinical decision-making process.

## References

[1] World Health O. Clinical management of severe acute respiratory infection when novel coronavirus (2019-nCoV) infection is suspected: interim guidance. Geneva: World Health Organization; 2020.

[2] China NHCotPsRo.Diagnosis and treatment protocols of pneumonia caused by a novel coronavirus (trial version 7). Beijing: National Health Commission of the People’s Republic of China; 2020.

[3] Huang C, Wang Y, Li X, Ren L, Zhao J, Hu Y, et al. Clinical features of patients infected with 2019 novel coronavirus in Wuhan, China. Lancet 2020; 395: 497–506. doi: 10.1016/S0140-6736(20)30183-5 31986264 PMC7159299

[4] Song F, Shi N, Shan F, Zhang Z, Shen J, Lu H, et al. Emerging 2019 novel coronavirus (2019-nCoV) pneumonia. Radiology 2020; 295: 210–7. doi: 10.1148/radiol.2020200274 32027573 PMC7233366

[5] Fang Y, Zhang H, Xu Y, Xie J, Pang P, Ji W. Ct manifestations of two cases of 2019 novel coronavirus (2019-nCoV) pneumonia. Radiology 2020; 295: 208–9. doi: 10.1148/radiol.2020200280 32031481 PMC7233358

[6] Chung M, Bernheim A, Mei X, Zhang N, Huang M, Zeng X, et al. Ct imaging features of 2019 novel coronavirus (2019-nCoV. Radiology 2020; 295: 202–7. doi: 10.1148/radiol.2020200230 32017661 PMC7194022

[7] Bernheim A, Mei X, Huang M, Yang Y, Fayad ZA, Zhang N, et al. Chest CT findings in coronavirus Disease-19 (COVID-19): relationship to duration of infection. Radiology 2020; 295: 200463. doi: 10.1148/radiol.2020200463 32077789 PMC7233369

[8] Qian L, Yu J, Shi H. Severe acute respiratory disease in a Huanan seafood market worker: images of an early casualty. Radiology 2020; 2: e200033. 10.1148/ryct.2020200033 PMC7233436 33778546

[9] Pan F, Ye T, Sun P, Gui S, Liang B, Li L, et al. Time course of lung changes on chest CT during recovery from 2019 novel coronavirus (COVID-19) pneumonia. Radiology 2020; 0: 200370. doi: 10.1148/radiol.2020200370 PMC7233367 32053470

[10] Ye Z, Zhang Y, Wang Y, Huang Z, Song B. Chest CT manifestations of new coronavirus disease 2019 (COVID-19): a pictorial review. Eur Radiol 2020; 30: 4381–9. doi: 10.1007/s00330-020-06801-0 32193638 PMC7088323

[11] van Griethuysen JJM, Fedorov A, Parmar C, Hosny A, Aucoin N, Narayan V, et al. Computational radiomics system to decode the radiographic phenotype. Cancer Res 2017; 77: e104–7. doi: 10.1158/0008-5472.CAN-17-0339 29092951 PMC5672828

[12] Yaniv Z, Lowekamp BC, Johnson HJ, Beare R. SimpleITK image-analysis Notebooks: a collaborative environment for education and reproducible research. J Digit Imaging 2018; 31: 290–303. doi: 10.1007/s10278-017-0037-8 29181613 PMC5959828

[13] Shu J, Tang Y, Cui J, Yang R, Meng X, Cai Z, et al. Clear cell renal cell carcinoma: CT-based radiomics features for the prediction of Fuhrman grade. Eur J Radiol 2018; 109: 8–12. doi: 10.1016/j.ejrad.2018.10.005 30527316

[14] Zhang B, Tian J, Dong D, Gu D, Dong Y, Zhang L, et al. Radiomics features of multiparametric MRI as novel prognostic factors in advanced nasopharyngeal carcinoma. Clin Cancer Res 2017; 23: 4259–69. doi: 10.1158/1078-0432.CCR-16-2910 28280088

[15] Bektas CT, Kocak B, Yardimci AH, Turkcanoglu MH, Yucetas U, Koca SB, et al. Clear cell renal cell carcinoma: machine Learning-Based quantitative computed tomography texture analysis for prediction of Fuhrman nuclear grade. Eur Radiol 2019; 29: 1153–63. doi: 10.1007/s00330-018-5698-2 30167812

[16] Shu J, Wen D, Xi Y, Xia Y, Cai Z, Xu W, et al. Clear cell renal cell carcinoma: machine learning-based computed tomography radiomics analysis for the prediction of WHO/ISUP grade. Eur J Radiol 2019; 121: 108738. doi: 10.1016/j.ejrad.2019.108738 31756634

[17] Bier G, Bier S, Bongers MN, Othman A, Ernemann U, Hempel J-M. Value of computed tomography texture analysis for prediction of perioperative complications during laparoscopic partial nephrectomy in patients with renal cell carcinoma. PLoS One 2018; 13: e0195270. doi: 10.1371/journal.pone.0195270 29668695 PMC5905959

[18] Krittanawong C, Zhang H, Wang Z, Aydar M, Kitai T. Artificial Intelligence in Precision Cardiovascular Medicine. J Am Coll Cardiol 2017; 69: 2657–64. doi: 10.1016/j.jacc.2017.03.571 28545640

[19] Venkatasubramaniam A, Wolfson J, Mitchell N, Barnes T, JaKa M, French S. Decision trees in epidemiological research. Emerg Themes Epidemiol 2017; 14: 11. doi: 10.1186/s12982-017-0064-4 28943885 PMC5607590

[20] Nie P, Yang G, Wang Z, Yan L, Miao W, Hao D, et al. A CT-based radiomics nomogram for differentiation of renal angiomyolipoma without visible fat from homogeneous clear cell renal cell carcinoma. Eur Radiol 2020; 30: 1274–84. doi: 10.1007/s00330-019-06427-x 31506816

[21] Lippi G, Plebani M. Laboratory abnormalities in patients with COVID-2019 infection. Clin Chem Lab Med 2020; 58: 1131–4. doi: 10.1515/cclm-2020-0198 32119647

[22] Pang J, Wang MX, Ang IYH, Tan SHX, Lewis RF, Chen JI-P, et al. Potential rapid diagnostics, vaccine and therapeutics for 2019 novel coronavirus (2019-nCoV): a systematic review. J Clin Med 2020; 9: 623. doi: 10.3390/jcm9030623 32110875 PMC7141113

[23] Jin Y-H, Cai L, Cheng Z-S, Cheng H, Deng T, Fan Y-P, et al. A rapid advice guideline for the diagnosis and treatment of 2019 novel coronavirus (2019-nCoV) infected pneumonia (standard version. Mil Med Res 2020; 7: 4. doi: 10.1186/s40779-020-0233-6 32029004 PMC7003341

[24] Xie X, Zhong Z, Zhao W, Zheng C, Wang F, Liu J. Chest CT for typical 2019-nCoV pneumonia: relationship to negative RT-PCR testing. Radiology 2020; 0: 200343. doi: 10.1148/radiol.2020200343 PMC7233363 32049601

[25] Ai T, Yang Z, Hou H, Zhan C, Chen C, Lv W, et al. Correlation of chest CT and RT-PCR testing for coronavirus disease 2019 (COVID-19) in China: a report of 1014 cases. Radiology 2020; 296: E32–40. doi: 10.1148/radiol.2020200642 32101510 PMC7233399

[26] Lippi G, Simundic A-M, Plebani M. Potential preanalytical and analytical vulnerabilities in the laboratory diagnosis of coronavirus disease 2019 (COVID-19. Clin Chem Lab Med 2020; 58: 1070–6. doi: 10.1515/cclm-2020-0285 32172228

[27] Guan W-jie, Ni Z-yi, Hu Y, Liang W-hua, Ou C-quan, He J-xing, et al. Clinical characteristics of coronavirus disease 2019 in China. N Engl J Med Overseas Ed 2020; 382: 1708–20. doi: 10.1056/NEJMoa2002032 PMC7092819 32109013

[28] Chen N, Zhou M, Dong X, Qu J, Gong F, Han Y, et al. Epidemiological and clinical characteristics of 99 cases of 2019 novel coronavirus pneumonia in Wuhan, China: a descriptive study. Lancet 2020; 395: 507–13. doi: 10.1016/S0140-6736(20)30211-7 32007143 PMC7135076

[29] Li Q, Guan X, Wu P, Wang X, Zhou L, Tong Y, et al. Early transmission dynamics in Wuhan, China, of novel coronavirus-infected pneumonia. N Engl J Med 2020; 382: 1199–207. doi: 10.1056/NEJMoa2001316 31995857 PMC7121484

[30] Lei J, Li J, Li X, Qi X. Ct imaging of the 2019 novel coronavirus (2019-nCoV) pneumonia. Radiology 2020; 295: 18. doi: 10.1148/radiol.2020200236 32003646 PMC7194019

[31] Li Y, Xia L. Coronavirus disease 2019 (COVID-19): role of chest CT in diagnosis and management. AJR Am J Roentgenol 2020; 214: 1280–6. doi: 10.2214/AJR.20.22954 32130038

[32] Feng D-Y, Zhou Y-Q, Xing Y-F, Li C-F, Lv Q, Dong J, et al. Selection of glucocorticoid-sensitive patients in interstitial lung disease secondary to connective tissue diseases population by radiomics. Ther Clin Risk Manag 2018; 14: 1975–86. doi: 10.2147/TCRM.S181043 30349276 PMC6188005

[33] Zhang R, Zhu L, Cai Z, Jiang W, Li J, Yang C, et al. Potential feature exploration and model development based on 18F-FDG PET/CT images for differentiating benign and malignant lung lesions. Eur J Radiol 2019; 121: 108735. doi: 10.1016/j.ejrad.2019.108735 31733432

[34] ACR recommendations for the use of chest radiography and computed tomography (CT) for suspected COVID-19 infection. 2020. Available from: https://www.acr.org/Advocacy-and-Economics/ACR-Position-Statements/Recommendations-for-Chest-Radiography-and-CT-for-Suspected-COVID19-Infection [March 11, 2020].

[35] Jacobi A, Chung M, Bernheim A, Eber C. Portable chest X-ray in coronavirus disease-19 (COVID-19): a pictorial review. Clin Imaging 2020; 64: 35–42. doi: 10.1016/j.clinimag.2020.04.001 32302927 PMC7141645

[36] Kong WF, Argarwal P. Chest imaging appearance of COVID-19 infection. Radiology: Cardiothoracic Imaging 2020; 2: e200028. doi: https://pubs.rsna.org/doi/abs/10.1148/ryct.2020200028 33778544 10.1148/ryct.2020200028 PMC7233424

